# A Review of Nanotechnology for Targeted Anti-schistosomal Therapy

**DOI:** 10.3389/fbioe.2020.00032

**Published:** 2020-01-31

**Authors:** Tayo Alex Adekiya, Pierre P. D. Kondiah, Yahya E. Choonara, Pradeep Kumar, Viness Pillay

**Affiliations:** Wits Advanced Drug Delivery Platform Research Unit, Department of Pharmacy and Pharmacology, School of Therapeutic Science, Faculty of Health Sciences, University of the Witwatersrand, Johannesburg, South Africa

**Keywords:** schistosomiasis, nanoparticles, drug delivery, targeted agents, molecular receptors, antibody, aptamers

## Abstract

Schistosomiasis is one of the major parasitic diseases and second most prevalent among the group of neglected diseases. The prevalence of schistosomiasis may be due to environmental and socio-economic factors, as well as the unavailability of vaccines for schistosomiasis. To date, current treatment; mainly the drug praziquantel (PZQ), has not been effective in treating the early forms of schistosome species. The development of drug resistance has been documented in several regions globally, due to the overuse of PZQ, rate of parasitic mutation, poor treatment compliance, co-infection with different strains of schistosomes and the overall parasite load. Hence, exploring the schistosome tegument may be a potential focus for the design and development of targeted anti-schistosomal therapy, with higher bioavailability as molecular targets using nanotechnology. This review aims to provide a concise incursion on the use of various advance approaches to achieve targeted anti-schistosomal therapy, mainly through the use of nano-enabled drug delivery systems. It also assimilates the molecular structure and function of the schistosome tegument and highlights the potential molecular targets found on the tegument, for effective specific interaction with receptors for more efficacious anti-schistosomal therapy.

## Introduction

Schistosomiasis is recognized as the second most prevalent among the group of NTDs in sub-Saharan Africa, following hookworm infection (Adekiya et al., 2017). Schistosomiasis is an infectious disease caused by parasitic worms that belong to the group of trematode and genus of *Schistosoma*, that results in chronic and acute disease (Adekiya et al., 2017). It poses a significant challenge on agricultural productivity and the life, growth and development of pregnant women and school children in afflicted areas. The disease-causing species of *Schistosoma* are *Schistosoma mansoni*, *Schistosoma haematobium*, *Schistosoma japonicum*, *Schistosoma intercalatum*, and *Schistosoma mekongi* (Adekiya et al., 2017; da Paixão Siqueira et al., 2017). For these worms to cause disease, the intermediate hosts (freshwater snails) need to be infected with the miracidia in freshwater where it develops into cercaria. Following human-water exposure, the cercaria penetrates the intact skin of humans.

Schistosomiasis affects the world’s poorest countries where there is no safe water, basic sanitation and hygiene education (da Paixão Siqueira et al., 2017). Currently, over 200 million people have been affected by schistosomiasis, including 40 million women of reproductive age and approximately 600–779 million individuals are at risk of becoming infected. The mortality rate has been estimated at 280,000 deaths annually in Sub-Saharan countries (Cioli et al., 2014).

The parasitizing of this infectious disease results in fever, malaise, abdominal pain, and skin rashes in an acute state, while intestinal, liver, urinary tract and lung diseases are the result of chronic infection. Acute and chronic disease is solely reliant on the type of species that infects an individual. Reappearance of schistosomiasis over latent periods can result in blockage of the urinary tract and pulmonary hypertension that can lead to fatal complications. In addition, schistosome infection promotes the severity of infection with additional pathogens such as; *Plasmodium falciparum, Toxoplasma gondii, Leishmania* spp., *Mycobacteria, Staphylococcus aureus, Salmonella*, and *Entamoeba histolytica* (Abruzzi and Fried, 2011).

The incidence of schistosomiasis is predominant in Sub-Saharan Africa, and with the increasing rate of infection, due to climate change and other socio-economic factors. To date, PZQ remains the only drug for the treatment of this debilitating disease. PZQ has the following benefits: (1) its effective against all forms of Schistosomes, (2) it is inexpensive and readily available and (3) it has a low side-effect profile, well tolerated in patients of all ages. Unfortunately, the use of PZQ is limited by the following: (1) drug resistance, (2) poor patient compliance to treatment in certain populations, (3) its ineffective against immature forms of the *Schistosoma* species and (4) it cannot prevent re-infection of Schistosomiasis. Furthermore, there is an increase in parasite alteration and modification, the global parasite load and co-infection with several strains of *Schistosoma* parasites (Caffrey, 2007; Doenhoff et al., 2008; Fenwick et al., 2009). Coupled with cases of cerebral schistosomiasis in some regions globally, there is an urgent need for an alternative anti-schistosomal drug molecule or to improve the delivery efficacy of PZQ using approaches such as nanotechnology to achieve targeted anti-schistosomal therapy, for example in the CNS.

**FIGURE 1:**
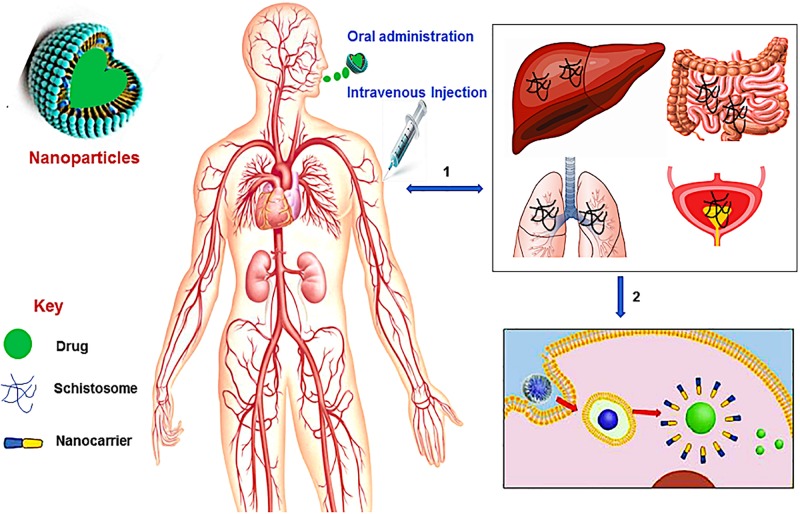
A schematic overview of nanotechnology in schistosomiasis treatment. **(1)** The Schistosoma parasites penetrates the human skin and enter the bloodstream where they travel via the blood vessels of the liver and lungs, and then to the vein around the intestines and bladder, **(2)** the administration (oral or intravenous injection) of nanotechnological-based drug leads to the disruption of the membrane (tegument) of the worms thereby releasing the drug to kill the worms.

There has not been a considerable impetus placed on developing novel and new drug treatments for schistosomiasis. However, based on the debilitating impact of the disease, researchers need to be alerted on exploring several essential target proteins found in the *Schistosoma species* and could play a significant role in ensuring the possibility of designing new drug molecules for schistosomiasis (Garcia-Salcedo et al., 2016). In the absence of any meaningful drug discovery programs for identifying new drug targets and molecules for schistosomiasis, pharmaceutical researchers have turned to providing more efficacious delivery systems for the gold-standard drug PZQ. Hence, nanotechnology and the use of nano-enable drug delivery systems (Figure 1), has been a major focus to potentially provide better treatment outcomes for schistosomiasis using PZQ (Veerasamy et al., 2011). Nano-enabled drug delivery systems can enhance the bioavailability and therapeutic efficacy of PZQ (or other drugs) and reduce the side effect profile by having more targeted drug delivery. Nanoparticulate systems currently researched involve, but are not limited to, lipid-based nanoparticles (liposomes, micelles, solid lipid nanoparticles, nanostructured lipid carriers and nanodiscs). Others include polymeric-based nanoparticles (nanospheres, nanocapsules, nanofibers/nanotubes, nanodiscs and micelles), metallic/inorganic-based nanoparticles (nanospheres, nanocapsules, nanodiscs and nanowires/nanotubes) and metal nanoparticles; fabricated by green chemistry (gold, silver, copper, platinum, palldium and zinc nanoparticles).

**FIGURE 2:**
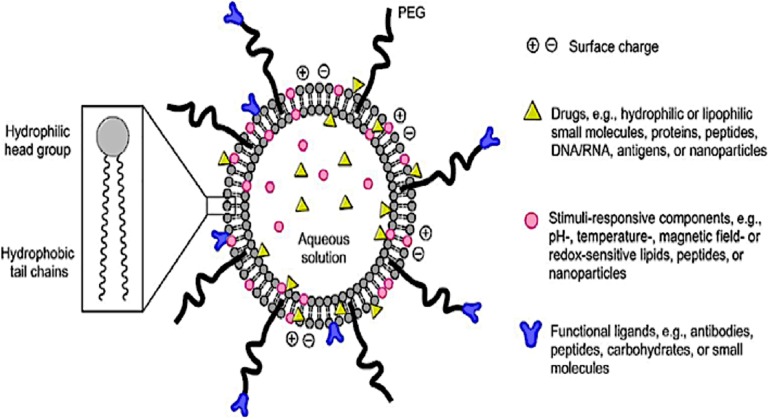
A schematic diagram of a smart lipid-based nanoparticles system as a nano-enabled drug delivery platform (reprinted with permission from Li and Takeoka, 2018).

The emergence of smart LBNPs (Figure 2) has offers secure platforms for the use of nano-biomaterials in medical applications such as encapsulation of therapeutic drugs for the targeted delivery of drugs for the treatment of diseases in biomedicine. Recently, the use of LBNPs has gained much interest, particularly in treating schistosomiasis, due to a better absorbed tegument of the schistosomes, which has an affinity for the phospholipid bilayer. LBNPs amphipathic nature allows them to play a pivotal role in the solubility modification and rate at which drugs such as PZQ can be targeted, for enhancing drug absorption across biological barriers (Cheng et al., 2017). Furthermore, targeted LBNPs can improve the efficacy and specificity of drugs to cells or tissues by upregulating surface molecular receptors such as antigens, unregulated selectin and serpin enzyme complex-receptor (Cheng et al., 2017). The *Schistosoma* parasite consists of different molecules that are found on the surface of the parasite tegument, which are needed for the parasite survival. This is a largely unexplored approach for targeted drug delivery in anti-schistosomal therapy. To this end, nanotechnology has played a central role in the design of systems intended to target the parasite tegument. Hence, this review aims to provide a concise incursion into the molecular structure and function of the schistosome tegument and assimilate the potential targeting proteins/molecules on the tegument to identify new targets and targeting molecules in anti-schistosomiasis therapy.

## Overview of the Past and Present Anti-Schistosomiasis Therapies

In 1984, the WHO Expert Committee proposed chemotherapy as the best treatment approach to eliminate schistosomiasis (Conlon, 2005). Ever since, chemotherapy continues to be the only measure for the control of schistosomiasis and depends only on a single dose treatment with PZQ. Among other anti-schistosomal drugs that have been explored, PZQ is the most widely used. PZQ is active against all forms of *Schistosoma* species that cause schistosomiasis. It reduces the parasitic load and is able to reduce the severity of symptoms. It is also the most preferred drug because of its simple administration, efficacy and affordability. Although, the mechanism of action in treating schistosomiasis is not well understood, a widely proposed mechanism is the immediate alteration in the worm musculature. This was reported by Pax et al. (1978) when they noticed that the alteration in the worm musculature causes contraction probably due to rapid influx of Ca^2+^ into the schistosome. This assertion was corroborated by interesting work undertaken by Kohn et al. (2001) that drew attention to the voltage-gated calcium channels of schistosomes as the potential target for PZQ. In their study, the mechanism of action for PZQ was suggested to be consistent with the observed effects of PZQ on Ca^2+^ homeostasis in schistosomes. It was noted that β-subunits of schistosome channels had a unique form of β-subunit structure that was different from other common β-subunits which inhibit flow of current through the α_1_ subunit of schistosome with which they are associated. The study further hypothesized that PZQ facilitated the opening of more channels for current to flow leading to the disruption of α1/β interaction in these channels resulting in disruption of Ca^2+^ homeostasis (Kohn et al., 2001). It has also been reported that PZQ causes morphological transitions in the schistosomes tegument. This was initially indicated by the formation of vacuoles within the tegument and blebbing at the surface (Becker et al., 1980; Mehlhorn et al., 1981; Cioli and Pica-Mattoccia, 2003). These morphological transitions cause increased exposure of antigens on the surface of the parasite (Harnett and Kusel, 1986). Harnett and Kusel suggested that the action of PZQ on the exposed antigens may be due to its lipophilicity that makes it easier to interact with hydrophobic cores of the tegument.

Due to the shortcomings of the drugs listed in Table 1, researchers have resorted to the use of drug delivery technologies such as nanotechnology to provide more targeted therapies to all stages of the Schistosoma parasite such that drugs can be more effective in treating the immature forms of the parasite. These novel approaches can also reduce drug resistance and avert re-infection by clearing the schistosomes in the human host.

**TABLE 1:** Drugs that have been used to treat schistosomiasis to date, with their shortcomings evaluated.

**Anti-schistosomal drugs**	**Shortcomings**	**Reference**
Metrifonate	Metrifonate is selective to only *S. haematobium* and due to medical standards and economic operations, the drug has been withdrawn from the market.	Eissa et al., 2011; de Moraes, 2012; Aruleba et al., 2018
Oltripaz	Oltripaz is another anti-schistosomal drug which has been used in the past, but not in the market again and discontinued in treating schistosomes infection due to its photosensitivity induction and longer time in curing the infections; approximately 2 months.	Nare et al., 1992
Niridazole	Niridazole was jettisoned due to its unpleasant adverse effects which include non-specificity destruction to the T waves electrocardiogram (ECG), toxicity to the renal and central nervous system, it has also been revealed to be a carcinogenic material.	Urman et al., 1975; Katz, 1977; Nare et al., 1992; Thetiot-Laurent et al., 2013
Oxamniquine	Oxamniquine has also been used in the past, but it is ineffective against all schistosomes type, only effective to *S. mansoni*, and due to cost effectiveness, drug resistance and some side effects, the drug has been replaced by praziquantel in treating schistosomiasis.	Saconato and Atallah, 2000; Morgan et al., 2001; Richter, 2003; El Ridi and Tallima, 2013; Aruleba et al., 2018

## The Schistosome Tegument: Revisit of the Molecular Structure and Function for Targeted Drug Delivery

**FIGURE 3:**
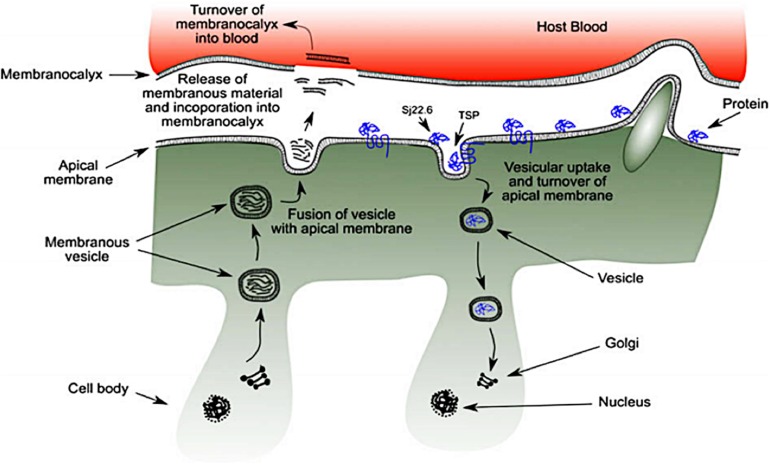
Schematic diagram of schistosome tegument structure with several organelles and the position of targeted proteins (as seen in blue) (reprinted with permission from Mulvenna et al., 2010).

The outer-surface of the schistosome is enclosed with an uncommon structure known as the tegument where some probable receptors for targeted nano-delivery system are found. It is a rare double layered membrane structure that plays a pivotal role in protecting the worm from harsh conditions in the host system. There are several organelles present in the tegument (Figure 3). The heptalaminate tegumental surface is enclosed by a typical plasma membrane structure that is superimposed by a secreted membranocalyx (generated by the multi-laminate vesicles found in the tegument cytoplasm) and fuses with lateral channels protruding out into the base of the surface from the cytoplasm which also host some potential proteins for nano-delivery systems. The membranocalyx can be active by interacting with proteins and glycans via the extracellular loops of the tetraspanins protein, this depicts tetraspanins as a probable target for nano-delivery systems.

The initiation of heptalaminate membrane surface alongside dyneins protein starts from the outer membrane of the cercarial trilaminate 30 min after invasion of cercarial into the host skin, and within 3 h, the change in cercarial membrane from the trilaminate to the heptalaminate mature membrane structure is accomplished in an immature schistosome (schistosomulum) (Mansour and Mansour, 2002), with the help of several molecules which are potential target for nano-delivery systems. The surface spines of schistosomes are made-up of paracrystalline arrangements of filamentous actin, which are found outside the tegument and basal membranes with protruding tip which is above the general level of the tegument. The dorsal surface spines protrude into the endothelial cells surface of the human host blood vessels with the help of different molecular proteins such as; dyneins, SGTP4 and tetraspanins (some of the potential targets for nano-enabled drug delivery systems), where it helps the schistosomes to hold fast against the blood flow when the schistosomes are living in the host mesenteric blood vessels (Kašný et al., 2017).

The syncytium of the tegument consists of trilaminate vesicles that comprise membranous material, and they are either elongated or spherical in shape and are known as membranous bodies and elongate bodies. The syncytium is linked to nucleated cell bodies (cytons) by cytoplasmic tubes that are coated with microtubules. Although, cytons are not considered to be part of the tegument, they are found under the circular and longitudinal muscle fibers of the tegument where some potential targeted proteins and molecules are secreted. They consist of mitochondria, nuclei, Golgi complexes, ribosomes and glycogen particles (Mansour and Mansour, 2002). The biogenesis of new membrane material including tegumental proteins, and the maintenance of the schistosomes tegument are equipped inside the cytons, under the muscular layer and syncytium vesicles (El Ridi et al., 2017). Though, the process has not been well studied, both the elongated bodies and membranous bodies transport membranous material which are found to be scattered in the cytons in conjunction with Golgi complexes where they are possibly generated. These vesicles carry membranous material and some targeted proteins produced within the cytons and move them via the tubules of the cytoplasm to the syncytium, and thereafter, migrate to the outside of the surface tegument of the membrane (El Ridi et al., 2017; Gobert et al., 2017; Kašný et al., 2017). More so, the incorporation of membrane vesicles content with other tegumental proteins (potential targeted molecules for nano-delivery systems) take place in the cytons where a fresh heptalaminate membrane is produced. This activity is not only restricted to schistosomula developments, also to adult schistosomes after the shedding or the rupture of the exterior schistosomes surface. Surface pits is another organelle found on the surface of the schistosomes tegument. The pits have an ability to increase the surface area of the worm not less than tenfold where it provides an avenue for the worm to absorb nutrients such as glucose and other molecules from the exterior milieu using its tegumental proteins and receptors.

Wendt et al. (2018) developed a new fluorescent schistosome tegument label technique. This proved that the schistosome parasite usually repairs and replaces the tegument continuously with a half-life of 5 days in order to survive harsh conditions in the host system (Wendt et al., 2018). This corroborated with the work of Wilson and Barnes (1977), where they showed that there is a possibility for the membranocalyx of the schistosome tegument to replace itself at a variable rate and was dependent on the external environmental conditions in which the worm was found. This view was supported by Perez and Terry (1973) where they observed that the surface of the schistosome was turning over more rapidly when schistosomes cultured in monkey anti-mouse serum were selected from the schistosome mice model.

This unique membrane structure allows the schistosome tegument to play a significant role in protecting the schistosome to survive in the host, some of which include; host response modulation that causes host immune response evasion (Elzoheiry et al., 2018). This immune evasion occurs by rendering the infected host’s antibody responses ineffective, hence fails to clear the established parasites (Han et al., 2009). In addition, the tegument of the schistosomes has some other functions such as absorption of nutrients.

Trematodes have an incomplete digestive tract, and the *Schistosoma species* can survive under prolonged *in vitro* incubation in the absence of nutrient absorption within the intestine (Isseroff and Read, 1974; Popiel and Basch, 1984). Glucose absorption in trematodes is noticed during immature stages of the trematode’s life cycle, which lacks a developed intestine (Uglem and Lee, 1985). Both, immature and mature schistosomes rely mainly on plasma glucose from the host for energy. Physiological investigations demonstrated that the influx of glucose within the tegument occurred by a carrier-mediated process (Rogers and Bueding, 1975; Uglem and Read, 1975). Several enzymes function for the absorption of amino acids on the tegument (Skelly et al., 2014), and other enzymes, for instance, leucine aminopeptidase is absent in the gut. Cholesterol is also acquired by schistosomes from the host via the tegument where it is redistributed throughout the schistosomes body (Haseeb et al., 1985; Popiel and Basch, 1986).

In terms of parasite motility control, the mature *Schistosoma* species move based on several degrees of flow and confinement. Zhang et al. (2019) observed that movement mechanics of schistosomes may be an important factor for the specific morphological qualities of an adult male worm, some of which include tegument topography and the strength as well as the nature of its suckers. In addition, the regulation of osmotic and electrochemical gradients of the worms is also control by the tegument. Faghiri and Skelly (2009) revealed that the schistosomes tegument controlled the movement of drug molecules and water into the parasites. This highlighted the role of the tegument in the uptake of drugs and in the osmoregulatory control of the parasite. The tegument also controls the excretion of certain metabolic products like amino acids, lactate, NH_4_^+^ and H^+^ (Faghiri et al., 2010; Skelly et al., 2014).

## Potential Molecular Targets in the Schistosome Tegument

There are several targets which have been identified on the surface of the tegument (Table 2). These are essential for engineered drug-loaded nanoparticles to target SGTP1 and SGTP4 as well as AChE and a nicotinic type of acetylcholine receptor (nAChR) that are predominantly found on the surface of the male schistosomes tegument. Other major surface proteins found on the tegument that can be targeted include dynein, aquaporins and tetraspanins among others. These molecules located on the surface of the tegument can serve as major molecular targets for the design and development of novel drug molecules and vaccines against the Schistosoma parasite.

**TABLE 2:** Different potential targets found on the schistosomes tegument for conjugated nanoparticles and their functions.

**Potential targets**	**Functions**	**References**
Schistosome Glucose Transporters	Facilitate the uptake of glucose required for energy production in schistosomes directly from the host bloodstream.	Skelly et al., 1994, 1998, 2014; Skelly and Shoemaker, 2000; Krautz-Peterson et al., 2010
Acetylcholinesterase (AChE) and a nicotinic type of acetylcholine receptor (nAChR)	They maintain the schistosomes ion channels and nervous system, and they have glucose scavenging modulatory activity from the mammalian host blood.	Camacho and Agnew, 1995; MacDonald et al., 2014
Microtubule liked-proteins (dyneins, actin, tubulin and paramyosin)	Microtube liked-proteins can play a role in schistosomes mobility. Dyneins helps in the attachment and detachment of the adjacent membranous organelles along microtubules. Also, they are implicated in the assembling of spindle which are used for chromosome movement in mitosis.	Braschi et al., 2006a, b; Githui et al., 2009; Simanon et al., 2019
Aquaporins	They control the flow of water molecules in and out of the schistosomes. Some associates of aquaporins family also helps in metabolites (e.g., lactate) diffusion in and out of the cell. In other words, aquaporins control the osmotic regulation of the schistosomes.	Tsukaguchi et al., 1998, 1999; Braschi et al., 2006a, b; Gonen and Walz, 2006; Faghiri and Skelly, 2009; Faghiri et al., 2010
Tetraspanins	They play an essential role in maintaining the plasma membrane structure of the schistosomes where they interact with one another. Also, interacts with many others, particularly associate proteins such as, integrins, MHC and co-stimulatory molecules to generate a huge signal transduction complexes known as tetraspanin-enriched microdomains (TEMs).	Braschi et al., 2006a; Tran et al., 2010; Sotillo et al., 2015
Molecular chaperone (heat shock proteins 70, 16, and 60)	They help the schistosomes to withstand stress by inducing heat shock responses. They may likely also be responsible for the dramatic changes in niche environments of the earlier stages of intra-mammalian schistosomula development.	Braschi et al., 2006a, b; Van Hellemond et al., 2006; Sotillo et al., 2015, 2016, 2019
Enzymes (Esterases, carbonic anhydrase, Phosphodrolases, Thoredoxin peroxidase, Glyceraldehyde-3-phosphate dehydrogenase, protein disulfide isomerase, Glutathione S-transferase etc.)	Carbonic anhydrase is responsible for the hydration of CO_2_ released by schistosomes during respiration. Phosphodrolases facilitate the removal of phosphate groups from organic molecules so that both could enter into the schistosomes via the plasma membrane. All other enzymes found on schistosomes tegument contribute toward the survival of the schistosomes.	Braschi et al., 2006a; Van Hellemond et al., 2006; Mulvenna et al., 2010; Sotillo et al., 2015, 2016, 2019

### Glucose Transporters as a Potential Molecular Target for Nano-Delivery Systems

Several studies (Skelly et al., 1994, 1998, 2014; Skelly and Shoemaker, 2000; Krautz-Peterson et al., 2010) have shown that *Schistosoma* parasites rely on energy (glucose) to survive. Energy (glucose) is consumed by the tegument first and not by the intestinal cecum. Uptake within the tegument is facilitated by the glucose transporters found on the tegument (Skelly et al., 1994, 1998). Skelly et al. (1994) isolated and characterized three different cDNAs with predicted protein sequences that indicate a high degree of structural and sequence similarity to that of facilitated diffusion transporters in animals, bacteria and plants. It was discovered that two cDNAs encoded for two different glucose transporters in the tegument namely SGTP1 and SGTP4. In addition, the study described that the SGTP 1 and 4 genes are expressed in adult and larval female and male schistosomes to facilitate the uptake of glucose from the host. In another related study by Cabezas-Cruz et al. (2015) four glucose transporters were encoded in the *Schistosoma mansoni* genome and only two out of the four facilitated glucose diffusion. Their results further proposed that *Schistosoma mansoni* class 1 glucose transporters failed to carry glucose and that this function developed independently in the schistosomes-specific glucose transporter.

There is a dynamic difference from the glucose transport of the platyhelminthes-specific transporters of the schistosomes when compared with humans (Cabezas-Cruz et al., 2015). It has been shown that the sequence of SGTP1 and 4 are 60% similar. Zhong et al. (1995) used electron microscopy to map the various locations of the transporters on the tegument. They observed that localization of SGTP1 was at the basal lamina and to a lesser extent under the muscle cells. This may help in transporting free glucose inside the tegument into the interstitial fluids that paddle the interior organs of the parasite. It was also demonstrated that SGTP4 was evenly distributed on the dorsal and ventral surfaces of female and male teguments with an extraordinary structure of a double lipid bilayer (Zhong et al., 1995). The distinct location of SGTP4 on the outer tegumental membrane reveals that SGTP4 facilitated glucose transport into the parasite tegument from the host bloodstream (Skelly et al., 1998; Skelly and Shoemaker, 2001). In addition, SGTP4 is involved in the development of the free-living cercariae into schistosomula. Through maturation they satisfy the needs of the parasite for high glucose uptake as soon as they enter the host (schistosomula stage) and throughout adulthood (Skelly and Shoemaker, 1996; Skelly et al., 1998).

Thus, proposing SGTP proteins as a potential target for nano-delivery systems, this postulation was supported by Krautz-Peterson et al. (2010) where RNAi was used to knock down the upregulation of SGTP4 and SGTP1 genes in schistosomula and in the life stages of adult worms. This study was undertaken to investigate the significance of these proteins to the parasite. Downregulation of either SGTP4 or SGTP1 displayed impairment in the ability of the protein to transport glucose when compared with the control. The study further showed that the simultaneous downregulation of both SGTP1 and SGTP4 reduced the ability of the parasite to transport glucose when compared with a single downregulated SGTP gene. It was also demonstrated that none of the parasites exhibited phenotypic distinction after prolonged incubation of all the suppressed parasites in enriched medium when compared to the control. Finally, it was suggested that SGTP1 and SGTP4 were important for transporting exogenous glucose from the mammalian host for normal parasite development. This was based on the observation that parasites with suppressed SGTPs showed decrease viability *in vivo* after infection of experimental animals (Krautz-Peterson et al., 2010). This notion was supported by a study performed by McKenzie et al. (2017), where the uptake of glucose was regulated in *Schistosoma mansoni* by Akt/Protein kinase B signaling. It was observed that Akt can be triggered by the host L-arginine, more so, insulin was shown to be effective in the layer of adult and schistosomula teguments. The inhibition of Akt decreased the upregulation and development of SGTP4 at the exterior of the host-invading larval stage of the parasite. The suppression of the SGTP4 upregulation at the tegument in adult worms was associated with a decrease in glucose uptake.

Hence, the functionalization of nanoparticles with targeted agents (antibodies, aptamers, antibody-like ligands, peptides and small molecules) with high specificity to SGTP proteins may be a superior alternative to anti-schistosomal treatment to nano-enabled the delivery of anti-schistosomal drugs. In achieving the desired selectivity of drug delivery, nanotechnology has allowed researchers to design nanoparticulate systems and incorporate therapeutic drugs to acts as nanocarriers. This is due to the overexpression of receptor molecules (SGTP proteins) which can serve as docking/interacting sites for targeting potential therapeutic drugs. Theoretically, the therapeutic drugs can be concentrated in a specific site in organ and tissues by functionalizing drug-containing nano-delivery systems with ligands against the receptors. Thus, nano-delivery systems with ligands specific to SGTPs as a receptor can be a potential target for designing, developing and delivering of anti-schistosomal drug.

### Acetylcholine (nAChRs), AChE and Nicotinic Receptors; Possible Targets for Nano-Delivery Systems

Acetylcholine (ACh) is an essential neurotransmitter, both in invertebrates and vertebrates. The neuromuscular consequences of ACh are normally mediated by postsynaptic nAChRs due to their high-affinity for nicotine. Based on the structure of nAChRs, they belong to the Cys-loop LGIC superfamily (Albuquerque et al., 2009; MacDonald et al., 2014). nAChRs generate hetero and homo-pentameric structures that are arranged in a barrel shape around a central ion-selective hole. nAChRs in invertebrates are anion and cation-selective (Cl_2_) ACh-gated channels while in vertebrates nAChRs are cation-selective (Ca^2+^, Na^+^, K^+^) and facilitate excitatory responses.

Both the nicotinic type of the acetylcholine receptor (nAChR) and AChE are potential target for nano-enabled drug delivery system, because they are both found on the exterior surface of the tegument where they play an essential role in the schistosomes ion channels and nervous system (Mansour and Mansour, 2002; MacDonald et al., 2014). AChE and nAChR are predominantly found on the surface of adult male schistosomes. The adult female schistosomes usually lodges in the gynaecophoral canal of the male containing a lower number of these proteins. AChE has been shown to have glucose scavenging modulatory activity from the human host bloodstream. The uptake of glucose is controlled by the interaction of ACh with the nAChR and AChE on the surface of the tegument (Camacho and Agnew, 1995). It has also been discovered that the exposure of low concentrations of ACh to *S. haematobium* or *S. bovis* and not *S. mansoni* improved the uptake of the glucose by the parasites in the host blood. At higher concentrations of ACh, the uptake of glucose in the host by parasites was inhibited. This specificity between the nicotinic receptor and ACh was supported by showing the effect of α-bungarotoxin and D-tubocurarine as antagonists to ACh. Therefore, it is significant when instituting a nanotechnology approach to deliver antagonistic drug molecules to the binding sites of nAChR and AChE in order to inhibit their glucose scavenging activities from the host bloodstream.

### Dyneins as a Possible Molecular Targets for Nano-Delivery Systems

Dyneins is a protein that produces force and movement on microtubules for biological processes such as ciliary beating, intracellular transportation and cell division, it performs these functions through the help of ATP hydrolysis (Roberts et al., 2013). Dyneins could serve as a possible target for nano-delivery system in the treatment of schistosomiasis owing to its biological function in the survival of the parasite. Several studies have employed immunostaining to identify various microtubule related proteins inside the schistosomes tegument such as actin, tubulin, paramyosin, and dyneins. Studies have suggested that cytoplasmic dyneins may have a role to play in transporting of vesicles to the surface bilayers and tegument cytoplasm from the sub-tegumental cells. Dynein chains are part of a huge enzyme complex comprising heavy, intermediate and light chains. Dyneins are implicated in the assembly of spindles that are used for chromosome movement in mitosis. The upregulation of dyneins are involved in the developmental of *S. mansoni.* In addition, they are found in the schistosomula stage that occurs after the penetration of the intact skin of the host by the parasite and at the lung stage in adult worms. At this stage, early upregulation of the heptalaminate exterior membranes are exhibited. Meanwhile, dynein light chains are not found in the cercariae or ciliated miracidia (Hoffmann and Strand, 1996).

The dynein light chain protein discovered recently was shown to have high affinity to other proteins tegument with which they form highly complex associations. Another dynein light chain protein has been considered as a tegument antigen with the molecular weight of 20.8 kDa. Githui et al. (2009) investigated the normal motor constituents of vesicular transport present in the schistosomes tegument. The NCBI database blast search analysis recognized clones that are myosin and dynein light chains genes. After subjecting the genes of schistosome dynein to further analysis in the databases, they detected three dynein light chains families. They also observed that the Tctex family sequences of the dynein light chains are different significantly when compare to the mammalian homologs, Hence, could serve as probable drug/vaccine target against schistosomes infection. The three dynein light chains, *S. japonicum* dynein light chain-1, *S. mansoni* dynein light chain and SM10 studied via the immunolocalization of microtubule-related motor protein components show a specific and strong immunolocalization in the distal cytoplasm of the tegument (Kohlstädt et al., 1997; Yang et al., 1999). The tegument-associated protein of the *S. japonicum* which has 22.6 kDa displays similar localization arrangements (Li et al., 2000). In view of these aforementioned roles of dyneins in schistosomes survival, the delivery of targeted drug to localize and bind to dynein using nanotechnology approach will be a potential technique in eradicating schistosomiasis. Nanotechnology-based targeted delivery system functionalized with specific targeted molecules (antibodies, peptides, antibody-like molecule and aptamer) can recognize and selectively bind onto the dynein protein (receptors) on its active region thereby conferring targeted delivery.

### Aquaporins as a Potential Molecular Targets for Nano-Delivery Systems

Aquaporins is another promising target for the delivery of surface-engineered drug-loaded nanoparticles. They are small integral membrane proteins that are mostly upregulated in animal and plant kingdoms. Aquaporins consist of two short and six transmembrane helical segments that enclose cytoplasmic and extracellular vestibules linked by a narrow aqueous pore. They consist of several conserved motifs, and aquaporin monomers are assembled as tetramers in membranes, with every monomer working independently (Verkman, 2013). Aquaporins act as channels to selectively control the influx and efflux of water molecules within cells. Certain aquaporins allow the diffusion of metabolites in and out of the cell (Tsukaguchi et al., 1998, 1999; Gonen and Walz, 2006).

The nano-enabling drug delivery activity of aquaporins was corroborated by Braschi et al. (2006a) where a proteomic study of the schistosome tegument was described. The presence of aquaporins was revealed on the surface of the tegument which indicated that aquaporins assisted with the influx of water and solutes within the plasma membrane of the schistosomes. The tegument (*S. mansoni*) as an excretory organ was investigated by Faghiri et al. (2010), where they observed that aquaporins on the surface of the tegument acted as a lactate transporter. In addition, it was also shown that the aquaporin found on the tegument was competent in transporting mannitol, water, alanine and fructose, but not glucose. Their further analysis of the tegument using immunofluorescent and immune-EM suggested that the function of the tegument was far above the known ability as an organ of nutrient uptake, but rather, it also helped in excretion of waste metabolites (Faghiri et al., 2010). The study supported the notion that the tegument controlled the osmoregulation and drug uptake in parasites (Faghiri and Skelly, 2009). It was also shown that the existence of aquaporins on the tegument controlled the movement of water following the suppression of *S. mansoni* aquaporins with iRNAs (Faghiri and Skelly, 2009).

It has also been shown that aquaporin-4 (a homolog of aquaporins) enhanced the granulomatous response with an increase in the accumulation of macrophages and eosinophils around the *S. japonicum* eggs in the liver of the mice model. Similarly, the study showed that aquaporin-4 mice enhanced Th2, but decreased Th1 and Treg cell formation in *S. japonicum.*, This accounts for the improvements of the liver granuloma formation (Zhang et al., 2015). These findings collectively indicate that aquaporins may be a desirable target for anti-schistosomal therapy using high precision delivery of drug-loaded nanostructures.

### Tetraspanins as a Potential Molecular Target for Nano-Delivery Systems

Tetraspanins (TSPs) is a family of integral membrane proteins expressed by schistosomes, found in the exterior surface of the membrane of the schistosomes tegument. Braschi et al. (2006a) identified five tetraspanins in the schistosomes membrane surface, and the abundant components of these proteins are found in the tegument periphery. They speculated that the schistosome tetraspanins play an important role in the structure of the schistosomes plasma membrane, based on their analogy with other organisms (Braschi et al., 2006a). They also showed that, some tetraspanins are recognized more readily than others, and the concentrations and locations of only three biotinylated are suggested to vary within the surface of schistosomes tegument. The capacity of tetraspanins homologous interaction to generate a tetraspanin web may help scaffold organization in the lipid bilayer upon which there are assemblage of other proteins within the tegument. More so, the extracellular loops of the tetraspanins may provide platforms for gylcans and proteins which interact with the membranocalyx (Braschi et al., 2006a).

The functions of tetraspanins in the tegument of *S. mansoni* was investigated with the inhibition of the upregulation of Sm-tsp-1 and Sm-tsp-2 mRNAs using RNA. The ultrastructural morphology of mature schistosomes treated with Sm-tsp-2 dsRNA, show a thinner tegument and there is a visible formation of vacuoles on the schistosomes tegument. More so, schistosomula exposed to Sm-tsp-2 dsRNA showed a drastic thinner and extensive vacuolated tegument, and this morphological observation depends on failure of tegumentary invaginations (Tran et al., 2010). In another related study by Sotillo et al. (2015), it was reported that tetraspanins were found in biotinylated and unbound tegument tissues. It was also reported that tetraspanin-2 found in *S. mansoni* is essential for the formation of the schistosomes tegument and is a target of protective immunity in naturally resistant human and vaccinated mice. On the other hand, *S. mansoni* tetraspanin-1 are detected on the apical membrane of schistosomula. Tetraspanin-2 was found only in the unbound sample, which corroborates with other findings, which shows that the localization of tetraspanin-2 within the inner compartments of the schistosomes; relates with the exterior invaginations and vesicles in the tegument (Sotillo et al., 2015). Targeted nanocarriers has three main components that is; as a targeting moiety-penetration enhancer, an apoptosis-inducing agent and also, as a carrier. Therefore, the inhibition of tetraspanin by the means of nanotechnology-based approach will stop the interaction of glycans and proteins to the schistosomes membranocalyx, because, nanomaterials can preferentially accumulate in the parasite via tetraspanin in an active targeting mechanism thereby, release the encapsulated drugs in a regulated manner. This will provide the benefits of increasing the anti-schistosomal drug concentration and its therapeutic efficacy.

### Other Potential Molecular Targets for Nano-Delivery Systems

Several studies have used proteomic in identifying constituents found within the tegument of schistosomes which potential targets for nano-delivery systems are. Braschi et al. (2006a) used proteomics to detect molecules found within the *S. mansoni* tegument. In their study, they identified transporters for sugars, inorganic ions, amino acids and water, which indicated that the tegument plasma membrane was crucial for schistosomes to acquire nutrients from the host and help maintain solute levels. They also identified enzymes such as esterases, phosphohydrolases and carbonic anhydrase with their catalytic domains found in the outer core of the plasma membrane, more so, annexin, five tetraspanins and dysferlin were shown to play a pivotal role in the architecture of the membrane. The study was corroborated by another proteomic analysis of *S. mansoni* proteins that was performed in the same year by Braschi et al. (2006b) not less than fifty-one (51) proteins were identified based on homology with known proteins in other organisms. Some of the identified proteins were enolase which involves energy metabolism; several cytoskeletal and molecular motor proteins such as severin, actin and dynein light chains. Others include molecular chaperone heat shock proteins 17, 19, and 20, calmodulin; vesicle proteins, and plasma membrane transporters; mitochondrial proteins for example ATP synthase; structural molecules and enzymes such as glucose transporter protein, calcium ATPase, annexin, alkaline phosphatase and tetraspanins A, B, and C (Braschi et al., 2006b).

**FIGURE 4:**
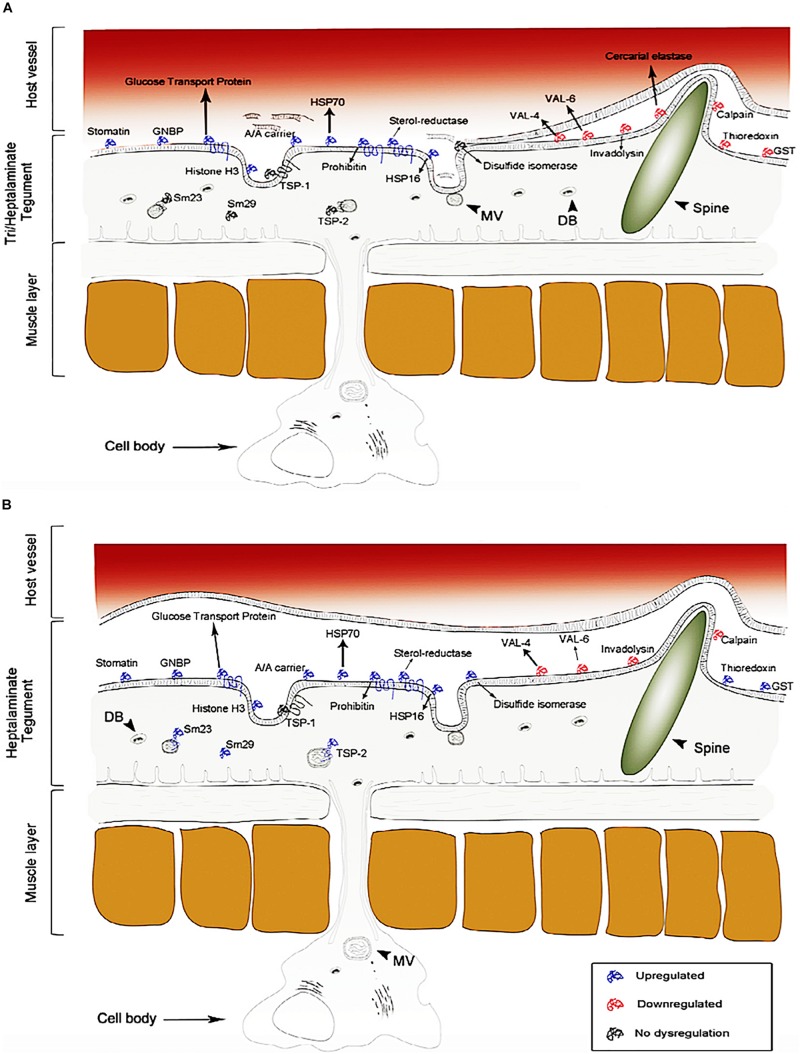
Proteome identification of upregulated, downregulated and no dysregulation proteins found on the tegument of *S. mansoni* (schistosomula). The dysregulation of these proteins changes over time. **(A)** 3 h of infection and **(B)** 5 days of infection (reprinted with permission from Sotillo et al., 2015).

As shown in Figure 4Sotillo et al. (2015) used the same approach to detect novel therapeutic targets for nanocarriers in *S. mansoni* schistosomula. Over 450 proteins were detected on the apical membrane of *S. mansoni* schistosomula, in which the expression of 200 have significant controlled profiles at diverse stages of schistosomula development *in vitro* which are potential targets for nano-delivery systems, such as glucose transporters, heat shock proteins, sterols, antioxidant enzymes and peptidases. In addition, current vaccine antigens were also detected on the apical membrane such as calpain, Sm-TSP-1 or Sm-TSP-2, Sm29 found on sub-tegumental fractions of the schistosomula showing localization patterns that differ in some instances from those found on the adult stage of the worm. Another study used S. mansoni genome project, concurrently with proteomic and lipidomic approaches, which allowed the study to characterize the lipids and proteins within the tegument plasma membrane in details. This study detected some tegumental targeted proteins and lipids, which depicts the role of the tegument in the uptake of nutrients from the host, and in the evasion of immune response. Furthermore, the study demonstrated that the tegument of the worm is enriched in lipids which are not found in the host. Similarly, the schistosome tegument possess proteins which have no sequence similarity with any other sequence found in databases of species excepts in schistosomes (Van Hellemond et al., 2006). Several other studies (Liu et al., 2006; Pérez-Sánchez et al., 2006; Mulvenna et al., 2010; Castro-Borges et al., 2011; Sotillo et al., 2016, 2019) have employed proteomics technique in identification of several molecular receptors which are druggable and vaccine candidates for schistosomiasis treatment.

To date, there is no effective vaccine for schistosomiasis. Although, several potential promising vaccine candidates for *S. mansoni* and, to a lesser extent, *S. haematobium* have been discovered and published in literature. There is one vaccine, namely, BILHVAX, or the 28-kDa GST from *S. haematobium*, which has entered clinical trials (Capron et al., 2005). Unfortunately, published data is not available on the clinical efficacy of this vaccine, but nonetheless, it is of concern that other vaccines have not progressed to this stage. More so, there are several nanotechnology approaches in developing vaccines for schistosomiasis published in literature, but have not entered clinical trials. Some include oral vaccination with chitosan nanoparticles loaded with plasmid DNA encoding a Rho1-CTPase protein of *S. mansoni* (Oliveira et al., 2012). Another approach includes a novel nanoparticle formulation of the Sj26GST DNA vaccine; although there was no significant reduction in worm burden, a highly significant decline in tissue egg burden and the fecundity of female adult worms was reported (Mbanefo et al., 2015).

## An Overview of Nano-Delivery Systems

**FIGURE 5:**
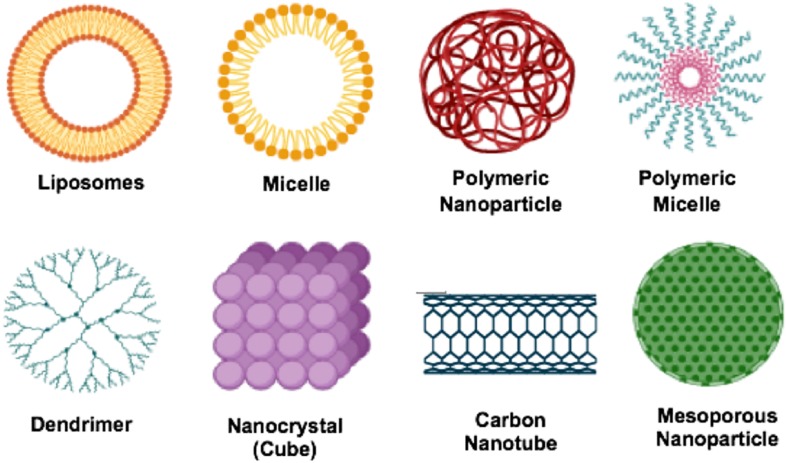
Types of nanocarriers for drug delivery.

Nanomedicine is the application of nanotechnology for treatment, prevention, monitoring, and control of biological diseases. In applying nanomedicine in the treatment of diseases, the precise targets (cells and/or receptors) specific to the clinical disease is identified and the suitable nanoparticles for the delivery system to minimize the side effects and improve the efficacy of the original drug is selected. One of these precise targets are macrophages, endothelial cells, proteins, dendritic cells as well as tumor cells. Some typical examples of nano-delivery systems (Figure 5) used over the years in the treatment of diseases includes; liposomes, micelles, dendrimer, polymeric nanoparticles, polymeric micelles, metallic nanoparticles, nanotubes and nanocrystals.

**FIGURE 6:**
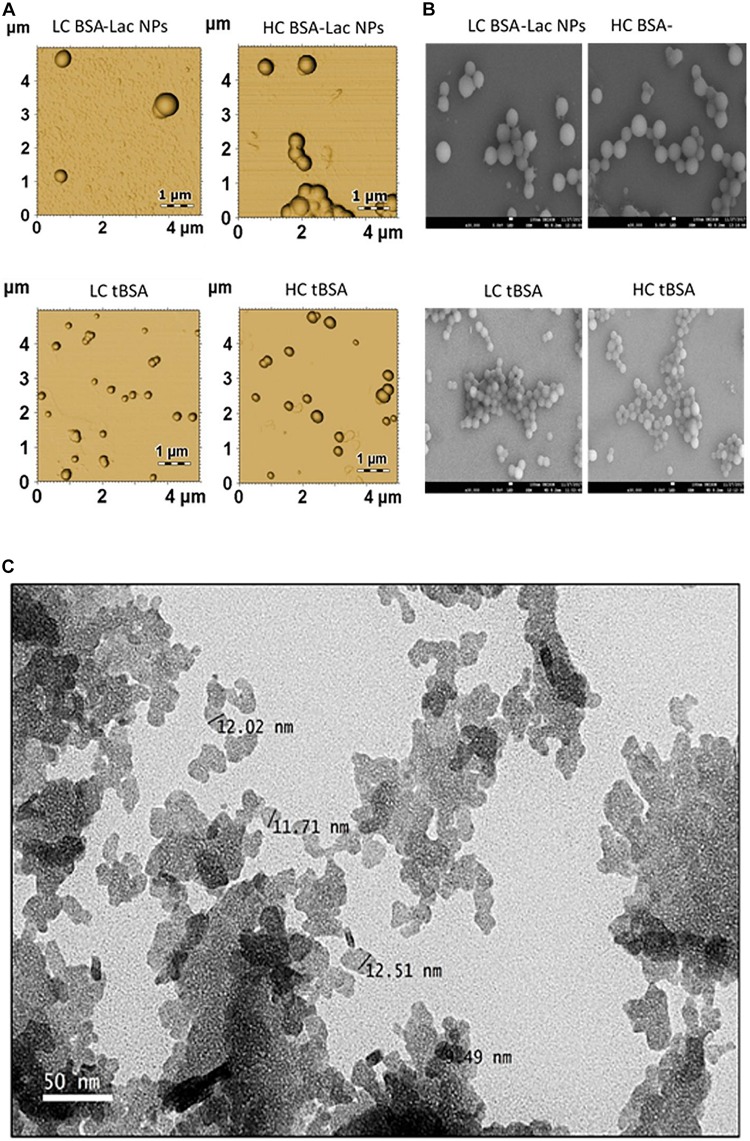
Morphology of HC and LC BSA-Lac and tBSA NPs **(A)** atomic force microscopy (AFM) and **(B)** Scanning electron microscopy (SEM). **(C)** Transmission electron microscopy (TEM) image of Citral-loaded self nano-emulsifying drug delivery system (CIT-SNEDDS). Adapted from Izham et al. (2019) and Teran-Saavedra et al. (2019).

Morphological characteristics such as rigidity, size and aspect ratio play a vital role in, and affect the impact and fate of nanocarrier properties *in vivo* (Wang et al., 2018). The properties of nano-delivery systems are critically dependent on the morphological characteristics of the particle, and it is of importance to deliver drug into a specific site during the treatment of disease, such as the delivery of an anti-tumor drug into the site of a solid tumor (Wang et al., 2018). The characterization of the nanoparticles morphology and dimensions can be determine using SEM, TEM, and AFM. Although, the most appropriate technique depends on the sample type and the desired information to be measured and in some cases, researchers usually adopt techniques which are available and well-known to them in a characteristic dimension of the nanoparticles. A typical example of TEM, SEM and AFM of nanoparticle are shown in Figure 6.

Several studies have explored the use of nano-delivery system in improving the therapeutic efficacy of different drug molecules in the treatment of diseases. Mehrizi et al. (2018) carried out the synthesis of a novel nanosized chitosan-betulinic acid delivery system, against resistant Leishmania, with the first clinical observation of this parasite in the kidney. It was discovered that chitosan nanoparticles synthesized using phase separation; and drug loading by phase separation, improved the therapeutic dose of betulinic acid to 20 mg/kg. More so, the successful improvement in the use of the nanosystem loaded betulinic acid in the treatment of leishmania, displayed both in *in vitro* and *in vivo* efficacy (Mehrizi et al., 2018).

The effectiveness of IVM was investigated using nanostructured lipid carriers in the treatment of hydatidosis, with some limitations and resistance associated with the drug overcome by the carriers in *in vitro* experimentation. It was observed that NLCs-loaded IVM induced higher mRNA caspase-3 expression which suggested a more potent apoptotic effect on the parasite (Ahmadpour et al., 2019). In another related study, nucleoside-lipid-based nanocarriers was used to encapsulate MB; a positively charged tricyclic phenothiazine molecule used in malaria treatment. This approach showed that the nanoparticles partially protected MB from oxido-reduction reactions, thereby preventing early degradation during storage, and the carrier also prolonged the pharmacokinetics in plasma. It was concluded that this approach was an interesting technique in improving MB stability and the delivery in malaria treatment (Kowouvi et al., 2019).

Hence, the utilization of lipid nanoparticle-based drugs in the treatment of schistosomiasis will be beneficial in terms of cost, since solid lipid nanoparticles are easy to scale-up and involves lower cost production, relative non-toxic nature, biodegradable composition and stability against aggregation. More so, lipid-based formulations have the ability to enhance the bioavailability of drugs through solubility modification and the rate at which drugs can be released for the improvement and enhancement of drug absorption across biological barriers (Cheng et al., 2017), reducing side-effects associated with these drugs. This type of approach will be beneficial and effective in treating all forms of schistosomes (both mature and immature), by functionalizing the nanoparticles with targeted molecules which has ability to recognize and bind to molecular receptors present in all forms of schistosomes. Thus, preventing reinfection by specifically targeting overexpressed schistosomes antigens present in the human host, it has been reported that nanoparticles have the ability to induce heightened T cell immunity, which can prevent disease reactivation and reinfection (Tousif et al., 2017). The list of various nano-delivery systems used in improving the therapeutic efficacy of PZQ in the treatment of *Schistosomal* infections are reported in Table 3.

**TABLE 3:** List of some nano-delivery systems which have been used in improving the therapeutic efficacy of PZQ in treating *schistosoma* infection.

**S/N**	**Test nanoparticles**	**Test *schistosoma* species**	**Efficacy**	**References**
1	PZQ-Liposomes	*S. mansoni*	Lip. PZQ causes a great significant reduction in the number of worm count, eggs/gram liver tissue and intestine. The nanosystem also reduced the number and diameter of hepatic granuloma in the histopathological studies.	Labib El Gendy et al., 2019
2	SLN-PZQ	*S. mansoni*	SLN-PZQ enhanced the bioavailability and antischistosomal efficacy against *S. mansoni* and reduced both the hepatic and intestinal tissue egg loads. In addition, the SLN-PZQ approximately cause complete disappearance on immature deposited eggs.	Radwan et al., 2019
3	Lipid nanocapsules (LNCs)-PZQ	*S. mansoni*	Oral LNCs-PZQ enhanced the efficacy of PZQ by targeting the distal postabsorption sites	Amara et al., 2018
4	Gold nanoparticles	*S. mansoni*	Gold NP showed to regulate gene expression impaired by *S. mansoni* infection	Dkhil et al., 2016
5	PZQ-Liposomes combined with hyperbaric oxygen therapy (HBO)	*S. mansoni*	100 mg/kg concentration of lip. PZQ + HBO was more effective (48.0% reduction of worms, 83.3% reduction of eggs/gram of feces) and 100% of the mice had altered of oograms; indicating interruption of oviposition. Additionally, HBO was able to stimulate the immune system, hence, HBO can work as an adjuvant in the treatment of the infection.	Frezza et al., 2015
6	PZQ-Liposomes	*S. mansoni*	There is improvement in the efficacy of the treatment with lip.PZQ, especially when administered 45 days following infection. More so, lip. PZQ is better absorbed by the tegument of *S. mansoni*, which has an affinity for phospholipids	Frezza et al., 2013
7	PZQ-Liposomes	*S. mansoni*	PZQ-liposomes caused a decrease in amounts of eggs and parasites. Liposomes improve the antischistosomal activity of praziquantel.	Mourão et al., 2005

## Targeted Nano-Enabled Drug Delivery

**FIGURE 7:**
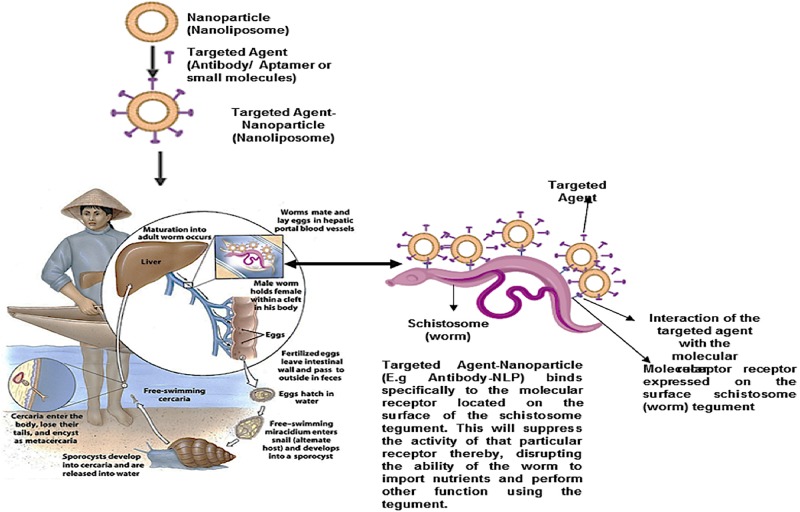
Proposed schematic for nanoparticle (nanoliposome) surface engineered (functionalized) with targeted agents (Antibodies, aptamers, antibody-like ligands or small molecules). This targeted nanoparticle localized/detected as molecular receptors, located within the exterior of the schistosome tegument, and specifically binds to it. Thus, suppressing the activity of the receptor, as well as disrupt the ability of the worm to import nutrients from the host, and perform other activities.

Targeted nano-enabled therapies are able to recognize or detect molecules that are highly expressed on the surface of specific cells. This approach has gained popularity in treating various cancers due to the overexpression of specific receptors on the membrane surface of cancer cells. In the field of cancer, targeted nanotherapies inhibit particular cell surface proteins or genes which are responsible for cancer growth and metastasis. It has been hypothesized that targeted nanotherapies may be desirable over other forms of treatment (Joo et al., 2013; Camidge, 2014). According to a 2018 review published by the ACS, targeted nanotherapies have been approved for various anti-cancer therapies. Thus, employing a nanotherapeutic approach to target overexpressed proteins or genes on the surface of the schistosome tegument will assist in overcoming PZQ resistant, reduce the burden of immature schistosomes (schistosomula), and finally, put an end to the morbidity and mortality of schistosomiasis (Figure 7). More so, this approach and can be employed in the treatment of various parasitic infections. Although there are no reports of targeted nano-enabled drug delivery against Schistosoma species to date; there are few reports of this type of approach on other similar parasites such as; the preparation of the primaquine-containing liposomes functionalized with covalently bound heparin for the targeted delivery of antimalarial drugs to pRBCs.

Heparin covalently linked with targeted nano-enabled drug delivery to pRBCs was carried out to reduce anticoagulation risks. The study showed that heparin-based targeting can be designed to have a greater half-life, further relying on antibodies with exposed antigens, whose expression is constantly modified by successive generations of the parasite (Marques et al., 2017). Further to this, targeted nano-enabled drug delivery of a 19-amino peptide from the circumsporozoite protein of *Plasmodium berghei* which contained a conserved region, as a consensus heparin sulfate proteoglycan-binding sequence attached to the distal end of a lipid-polyethylene glycol bioconjugated, was prepared by the incorporation into phosphatidylcholine liposomes, reflecting favorable *in vitro* results (Longmuir et al., 2006).

Jain et al. (2014) developed a chitosan-assisted immunotherapy for the intervention of experimental leishmaniasis via amphotericin B -SLPs to activate the macrophages in order to impart a specific immune response by improving the production of TNF-α and IL-12 (Jain et al., 2014). This study also reflected a positive hypothesis for targeted nano-drug delivery for site specific targeting.

### Antibody-Functionalized Drug Delivery Systems for Targeted Therapy in Schistosomes Infection

Antibodies are mostly IgGs or their fragments and have ability to recognize and interact virtually with any molecular target with high affinity and high specificity. Antibodies have gained special interest in targeted therapies due to their nanosized. They are biological materials which are part of the specific immune system, and they are toxin or pathogens neutralizers in nature. They help in recruitments of immune elements such as; improving phagocytosis, complement, cytotoxicity antibody dependent by NK cells. They can also help in carrying several elements such as; toxins, nanoparticles, drugs and fluorochroms to where they can be used in therapy to destroy a specific target, and for several other diagnostic procedures (Arruebo et al., 2009). Antibody-functionalized nanoparticulate systems are more site-specific, causing higher accumulation on the target region and subsequently, reduces dosage requirements.

The bioconjugation of antibodies with nanoparticles to generate a unique product which is composed of both the properties of the antibodies and nanoparticles can take place by adsorption process that is, at the isoelectrical point of the antibody through electrostatic interaction (Arruebo et al., 2009; Greene et al., 2018). More so, the conjugation can take place by direct covalent bonding between the surface of the antibody and the nanoparticles (that is, coupling of the antibodies to nanoparticles by free carboxyl or amine functionalities on aspartic/glutamic acid or lysine residues or by thio-maleimide reaction) (Arruebo et al., 2009). Another means by which the bioconjugation of the antibodies to the nanoparticles can be achieved is through the use of adapter molecules that is, bio-recognitions like streptavidin and biotin, which usually involves the formation of the complex. Greene et al. (2018), developed a novel approach for the site-specific conjugation of nanoparticulate systems which promotes the uniform and outward projection of paratopes for utmost target interaction. They demonstrated a successful re-bridging of the inter-chain disulfide linkage with a heterobifunctional linker and successive coupling to nanoparticles bearing complementary azide moieties in TRAZ F(ab) model. In a study by Mohammad et al., the administration of the antimalarial drug (chloroquine) loaded liposomes, targeted to infected RBCs with a tagged antibody against infected erythrocytes surface antigens on the chloroquine liposomes against drug-resistant *Plasmodium berghei*, presented a cure rate of 75–90% on days 4–6 post infection in mice (Mohammad et al., 1995).

Secret et al. (2013) described the preparation of antibody-functionalized biodegradable porous silicon nanoparticles loaded with the hydrophobic anticancer drug camptothecin. Using a novel semicarbazide based bioconjugation technique in chemistry, the specific orientation of the immobilized antibody on the nanoparticles was achieved. Three antibodies mAb528 a monoclonal antibody to EGFR; MLR2 a monoclonal antibody to p75NTR and Rituximab a monoclonal antibody to CD20 were used to target glioblastoma, neuroblastoma and B lymphoma cells, respectively in an *in vitro* study. The successful targeting was demonstrated by means of immunocytochemistry and flow cytometry both with cell lines and primary cells. The incubation of the antibody-functionalized nanoparticles with the cell lines for cell viability, showed selective killing of cells expressing the receptor, which correspond to the antibody coupled on the porous silicon nanoparticles. Also, the incorporation of camptothecin an anticancer drug into a nanoparticle functionalized with the antibodies showed to be very effective and efficient in targeting and killing cancer cells (Secret et al., 2013). In another recent study by Li et al. (2019), they developed an antibody-functionalized gold nanoparticle (cetuximab-AuNP) to selectively target cancer cells and probe for their potential radiosensitizing impacts under proton irradiation. It was discovered that cetuximab-AuNP interacts and bound specifically and accumulate in EGRF-overexpressing A431 cells when compared with EGFR-negative MDA-MB-453 cells. It was further shown that, cetuximab-AuNP improved the influence of proton irradiation in A431 cells but not in MDA-MB453 cells (Li et al., 2019). There are several other studies (Day et al., 2010; Dai et al., 2015; Korkmaz et al., 2016) which have employed antibody-functionalized nanoparticles to selectively target some specific receptors on the cancer cells either for treatments or imaging (diagnostics).

### Aptamer-Functionalized Drug Delivery System for Targeted Therapy in Schistosomes Infection

Aptamers are short single-stranded oligonucleotide (RNA or DNA ligands) or peptides that bind to their target molecules; either small chemicals, large molecular cell-surface or transmembrane proteins with high specificity, affinity and versatility. They have been developed for over two decades against several targets and for different applications. Aptamers have emerged as promising molecules to target specific cancer antigens in therapy and clinical diagnosis (Cerchia and De Franciscis, 2010; Catuogno et al., 2016). Nucleic acid aptamers have gained attention as an attractive molecular vehicle because of their ability to bind to specific ligands with high affinity, they have high ability to penetrate cells, tissues and organs, and they also possess high chemical flexibility (Catuogno et al., 2016). Whereas, peptide aptamers, otherwise known as affimers are small stable proteins that are selected to interact and attach with high binding affinity to specific sites (surface) on their target molecules. They contain short amino acid of about 5–20 residues long sequences which are normally embedded as a loop inside a stable protein scaffold (Reverdatto et al., 2015). Aptamer based sensing platforms for the recognition of peptides, small molecules, proteins and cells have gained a huge interest due to their high sensitivity and selectivity. In general, aptamers are molecules that can generate unique 3-dimentional structure and has the ability to bind almost any molecular targets with higher binding affinity in the nanomolar level compared to monoclonal or polyclonal antibody.

Due to aptamers properties such as; high affinity, chemical stability, small size, ease of synthesis, low-immunogenicity and controllable chemical modification, owing to these multiple attributes, aptamer conjugated nanoparticles are well qualified nanosystems for the development of biomedical devices for imaging, analytical, drug delivery and some other medical applications. The bioconjugation of the aptamers onto the nanoparticulate systems can be attained via non-covalent (affinities interaction e.g., streptavidin-biotin or metal ion co-ordination) and covalent (1-ethyl-3-carbodiimide or succinimdyl ester-amine chemistry and *N*-hydroxysuccinimide activation chemistry which cross link the carboxylic acid group on the surface of the nanoparticles and the amino group of the ligands) interactions. The covalent interaction strategies can also be achieved by maleimide-thiol chemistry that is, the cross linking of the thiol group on the targeting moiety and the maleimide functional group on the surface of the nanoparticulate systems.

Yu et al. (2011) developed a novel aptamer bioconjugated nanoparticles in order to enhance the delivery of paclitaxel anticancer drug to MUC1-positive cancer cells. The aptamer was engineered into the surface of the nanoparticles via a DNA spacer. The flow cytometry analysis shown the higher uptake of the nanoparticulate systems conjugated with MUC1 specific aptamer into the target cells via the overexpression of MUC1. The results further showed that, paclitaxel loaded aptamer functionalized nanoparticles improved the *in vitro* drug delivery and cytotoxicity to MUC1 cancerous cells when compared to non-targeted nanoparticulate systems which lack the MUC1 aptamer. In Yang et al. (2013) developed a DNA aptamer envelope protein for the inhibition of hepatitis C virus. In their study, it was shown that selected aptamers for E1E2 particularly recognized the recombinant E1E2 protein and E1E2 protein from hepatitis C virus-infected cells. The aptamers exert antiviral properties via the inhibition of the virus binding to the host cell (Yang et al., 2013). Several other studies (Mathieu et al., 2016; Corda et al., 2018; Dou et al., 2018; Tan et al., 2019) have employed aptamer-conjugate as a targeted delivery system for therapeutics and diagnostics.

### Other Functionalized Drug Delivery Systems for Targeted Therapy in Schistosomes Infection

Other small molecules or peptides that are highly specific for certain molecular receptors with high affinity, can also be screened or developed in order to localize and bind with the molecular receptors found on the tegument of the schistosomes. Lei et al. (2019) designed a novel alendronate-modified nanoparticle loaded with paclitaxel and coated with polydopamine for osteosarcoma-targeted therapy. In this study, it was reported that the polymerization of dopamine in a versatile modification method was not limited by the absence of functional groups on the surfaces of the compound and do not affect the chemical properties. The successful bioconjugation of the polydopamine with nanoparticles with a surface modifier which consist of a precise affinity for osteosarcoma cells was attained. They posit further that, the targeting nano-delivery systems exhibited a higher *in vitro* cytotoxicity on K7M2 of osteosarcoma cells when compared with the native nanosystems. Furthermore, the *in vivo* study showed that the targeting nano-delivery systems could accumulate within the tumor to a greater extent with remarkable decrease in the adverse effects of paclitaxel when compared with non-targeted nanosystems (Lei et al., 2019).

Säälik et al. (2019) investigated the effect of a novel penetrating peptide-guided nanoparticles that targets cell surface LinTT1, p32 for glioblastoma targeting. In this study, the coupling of LinTT1 to albumin-paclitaxel nano-delivery systems was achieved by sulfosuccinimidyl 4-(*N*-maleimidomethyl) cyclohexane-1-carboxylate as a linker. They demonstrated that the novel p32 targeting peptide, LinTT1 promotes the targeted accumulation of nanoparticles to tumors across a panel of high-grade glioma mouse model effectively. They further showed that the treatment of mice with LinTT1-guided nanoparticles extend the survival rate of mice with the tumor; due to the ability of LinTT1-nanopaticles to recognize the upregulation of p32 on glioblastoma (Säälik et al., 2019).

Ahlschwede et al. (2019) employed targeted nano-delivery approach in treating cerebral amyloid angiopathy and detecting cerebrovascular amyloid observed in AD. A targeted nano-delivery system was developed by a cationic blood brain barrier penetrating peptide using a covalent bioconjugation technique. The results from the targeted nanosystem depicted a higher significant brain uptake due to the high binding affinity of the peptide (K16ApoE)-nano-delivery system to amyloid plaques. In another study carried out by Colombo et al. (2019) where the targeted biodegradable nano-delivery system for CD34 + endothelial precursors in the treatment of rheumatoid arthritis was achieved. The bioconjugation of the targeting molecule was activated by *N*-hydroxysuccinimide in order to exploit its primary amino groups. The results in this study showed that, the targeted nano-delivery system possess a greater advantage in delivery the drug to inflamed synovia via the synovium-homing peptide as a targeting molecular receptor.

Silva et al. (2015) carried out mannose-functionalized polymeric nanoparticles to target the mannose receptors on antigen-presenting cells and therapeutic anti-tumor immune responses in a melanoma model. It was discovered that mannose-functionalized nanoparticles potentiated the Th1 immune activity, and the nanoparticulated vaccines reduced the rate of murine B16F10 melanoma tumors growth in prophylactic and therapeutic settings (Silva et al., 2015). Also, Mufamadi et al. (2013) carried out a ligand-functionalized nanoliposomes for targeted delivery of galantamine in AD. It was shown that ligand-functionalized nanoliposomes enhanced the uptake of galantamine into PC12 neuronal cells through the receptor of Serpin Enzyme Complex (Mufamadi et al., 2013). Ruff et al. (2017) investigated the effect of gold nanoparticles surface engineered with amyloidogenic β-amyloid specific peptides in a BBB in an *in vitro* model. This study was carried out in order to increase the BBB permeability, as well as the nanoparticle concentration in the brain by the peptides. It was discovered that, the multivalent peptides bind selectively to Aβ-amyloid fibrils, thereby posing a strongly effect on the integrity of BBB, thus, actively cause the transport of the gold nanoparticle conjugates via the BBB.

## Conclusion

Incidences of schistosomiasis continue to increase globally across sub-Saharan Africa and other tropical regions. However, the development of resistance against the only drug PZQ necessitates the design of more effective drug molecules to tackle the continual increase in Schistosomiasis cases. In this review, the molecular structure and function of the schistosomal tegument was described and several molecular targets have been identified to potentially target the schistosomes tegument as a site for enhanced PZQ delivery in anti- schistosomal therapy. In addition, potential agents that could target the molecular receptors identified have been highlighted. In general, surface functionalization of nanoparticles with antibodies, aptamers, antibody-like ligands, peptides and small molecules to specifically target and bind to the schistosomes tegument receptor genes and proteins presents a viable option for researchers to explore. This approach will suppress the activity of receptor genes/proteins, thereby impairing the ability of schistosomes to import nutrients from the host as well as disrupt the ability of the parasite to maintain solute balancing and evasion of the host immune response. Hence, exploration of the schistosomes tegument may be a possible and potential focus for designing and developing anti-schistosomal drug which can target receptors and proteins present on the worm tegument.

## Author Contributions

All authors contributed to the manuscript completion and approved the final submission. TA, PK, YC, PK, and VP contributed to this study from framework design and manuscript content to manuscript optimization.

## Conflict of Interest

The authors declare that the research was conducted in the absence of any commercial or financial relationships that could be construed as a potential conflict of interest.

## Abbreviations

Ach: acetylcholine
AChE: acetylcholinesterase
ACS: american cancer society
AD: alzheimer’s disease
AFM: atomic force microscopy
AuNP: gold nanoparticle
BBB: blood-brain barrier
CNS: central nervous system
IgGs: immunoglobulins
IVM: ivermectin
LBNPs: lipid-based nanoparticles
LGIC: ligand-gated ion channel
LNCs: lipid nanocapsules
MB: methylene blue
nAChR: nicotinic acetylcholine receptor
NK: natural killer
NLCs: nano-lipid carriers
NTDs: neglected tropical diseases
pRBCs: *plasmodium-*infected red blood cells
PZQ: praziquantel
RBCs: red blood cells
SEM: scanning electron microscopy
SGTP1: schistosome glucose transporter 1
SGTP4: schistosome glucose transporter 4
SLN: solid lipid nanoparticle
TEM: transmission electron microscopy
TSPs: tetraspanins
Th1: T-helper 1 cells
Th2: T-helper 2 cells
WHO: world health organisation.
